# Antimicrobial potential of *Streptomyces coeruleofuscus* SCJ isolated from microbiologically unexplored garden soil in Northwest Morocco

**DOI:** 10.1038/s41598-024-53801-x

**Published:** 2024-02-09

**Authors:** Said Rammali, Abdellatif Rahim, Mohamed El Aalaoui, Bouchaib Bencharki, Khadija Dari, Aicha Habach, Lamiri Abdeslam, Abdelkrim khattabi

**Affiliations:** 1 Laboratory of Agro-Alimentary and Health, Faculty of Sciences and Techniques, Hassan First University of Settat, B.P. 539, 26000 Settat, Morocco; 2 Laboratory of Biochemistry, Neurosciences, Natural Ressources and Environment, Faculty of Sciences and Techniques, Hassan First University of Settat, B.P. 539, 26000 Settat, Morocco; 3 Regional Center of Agronomic Research of Settat, Tertiary Road 1406, At 5 Km From Settat, 26400 Settat, Morocco; 4 Biotechnology Unit, National Institute of Agronomic Research of Rabat, Av. Annasr, 10000 Rabat, Morocco; 5 Applied Chemistry & Environment Laboratory, Faculty of Sciences and Techniques, Hassan First University of Settat, B.P. 539, 26000 Settat, Morocco

**Keywords:** *Streptomyces* sp., Antimicrobial activity, Multi-drug resistance, Phytopathogenic fungi, 16S rRNA gene, Volatile compounds, Antimicrobials, Antibiotics, Antifungal agents, Antimicrobial resistance, Biochemistry, Biotechnology, Microbiology

## Abstract

Research on microorganisms in various biotopes is required to identify new, natural potent molecules. These molecules are essential to control the development of multi-drug resistance (MDR). In the present study, a *Streptomyces* sp., namely SCJ, was isolated from a soil sample collected from a Moroccan garden. SCJ isolate was identified on the basis of a polyphasic approach, which included cultural, micro-morphological, biochemical, and physiological characteristics. The sequence of the 16S rRNA gene of the SCJ strain showed 99.78% similarity to strains of *Streptomyces coeruleofuscus* YR-^T^ (KY753282.1). The preliminary screening indicated that the SCJ isolate exhibited activity against *Candida albicans* ATCC 60,193, *Escherichia coli* ATCC 25,922, *Staphylococcus aureus* CECT 976, *Staphylococcus aureus* ATCC 25,923, *Bacillus cereus* ATCC 14,579, *Pseudomonas aeruginosa* ATCC 27,853, as well as various other clinical MDR bacteria and five phytopathogenic fungi. The ethyl acetate extract of the isolated strain demonstrated highly significant (p < 0.05) antimicrobial activity against multi-resistant bacteria and phytopathogenic fungi. The absorption spectral analysis of the ethyl acetate extract of the SCJ isolate obtained showed no absorption peaks characteristic of polyene molecules. Moreover, no hemolytic activity against erythrocytes was observed in this extract. GC–MS analysis of the ethyl acetate extract of the SCJ isolate revealed the presence of 9 volatile compounds including 3,5-Dimethylpyrazole, and pyrrolizidine derivatives (Pyrrolo[1,2-a]pyrazine 1,4-dione, hexahydro-3-(2-methylpropyl)), which could potentially explain the antimicrobial activity demonstrated in this study.

## Introduction

Antibiotics are considered miracle drugs applied in modern medicine. They have a crucial role in the treatment of pathogenic microorganism infections^1^. In 1928, Alexander Fleming accidentally discovered penicillin as the first natural antibiotic, however, it wasn't fully perfected for usage until the late 1930s^2^. Subsequently, additional microbiologists initiated a targeted exploration for antimicrobial agents within soil-derived fungi and bacteria^3^. Hence, this scientific success has allowed the greatest reduction in morbidity and mortality linked to infectious diseases^4^. Nevertheless, whether in the fields of medicine or agriculture, the incorrect and uninformed use of antibiotics resulting from their acquisition without expert consultation, has led to the emergence of multi-drug resistant (MDR) bacteria and fungi. These issues pose substantial and current challenges to public health^5,6^. Indeed, as demonstrated by multiple research studies, the phenomenon of multi-drug resistance (MDR) can give rise to complicated infections that pose significant challenges in terms of treatment, ultimately raising the risk of death^7,8^.

Recently, extensive studies have been focused on searching for microorganisms in various habitats to discover novel potent compounds with a large spectrum of biological properties^9–11^. Therefore, these investigations have revealed that *Actinobacteria***,** particularly the *Streptomyces* genus, exhibit various medicinal properties such as antimicrobial, antiparasitic, antioxidant, and anticancer effects, that have demonstrated efficacy against multiple pathogens and have gained widespread utilization for clinical applications. In Morocco, the Casablanca Settat region is primarily characterized by intensive agricultural practices involving the widespread use of fertilizers and pesticides^12^. In this study, our focus centers on the garden in Titt Mellil, chosen deliberately to investigate *Actinobacteria* thriving in untreated and unpolluted soils, thus minimizing the impact of synthetic chemicals. Therefore, the main objectives of the present study were to: (1) isolate *Actinobacteria* strains from a normal natural ecosystem (the Titt Mellil garden belonging to the Casablanca-Settat region of Morocco (33°33′14.1"N, 7°29′05.4"W)) and perform physicochemical analyses of the soil sample; (2) perform primary screening of the *Actinobacteria* isolate (SCJ strain) for antimicrobial activity; (3) perform morphological, biochemical and physiological characterization, as well as molecular identification by 16S rRNA gene sequencing of the *Actinobacteria* isolate; (4) determine the antimicrobial activity of the organic extract from the *Streptomyces* strain against selected multidrug-resistant (MDR) pathogenic bacteria and phytopathogenic fungi; (5) assess the toxicity of the organic extract using the UV–visible and hemolysis test (human red blood cells) techniques; 6) and determine the profile of compounds present in the ethyl acetate extract of the SCJ strain by gas chromatography (GC) coupled with mass spectrometry (MS).

## Results and discussion

### Physico-chemical analysis of soil sample

The production of secondary metabolites by *Actinobacteria* is integral to combating multidrug-resistant bacteria and phytopathogenic fungi^13^. However, this process is closely linked to abiotic factors, specifically the composition of soil in terms of OM, pH, EC, texture, and minerals^14^. Optimal levels of these parameters are pivotal in fostering microbial growth, thereby facilitating the biosynthesis of secondary metabolites^14^. Therefore, the physicochemical parameters of the soil were determined to unravel their impact on the physiology of *Actinobacteria*. In this study, physico-chemical analysis of this type of soil showed that it is a sandy-loam soil with a slightly alkaline pH, not salty, with normal levels of total nitrogen and organic matter. It is poor in mineral elements, with the exception of Si, O, Al, Ca and Fe, which are present in significant quantities (Supplementary Table S1, and Supplementary Fig. S1). *Actinobacteria* play a significant role in soil ecosystems. Their presence and function are influenced by several physico-chemical soil properties like pH, salinity, mineral composition, organic matter content, total nitrogen, and soil type^15^. The observed slightly alkaline pH is remarkable, as it positively impacts microbial activity by promoting nutrient availability through bacterial decomposition of organic matter^16^. Soil pH significantly affects soil microorganisms activity^17^, with alkaline soils being more desirable as they promote nutrient availability by facilitating bacterial decomposition of organic matter. This finding aligns with the results of Makut and Owolewa^18^, who reported that *Actinobacteria* flourish more effectively in alkaline soils. *Actinobacteria* have diverse pH preferences, with certain species thriving in slightly acidic soils, while others prefer neutral or slightly alkaline environments. Additionally, specific *Actinobacteria* species are adept at solubilizing important mineral elements in the soil, such as iron and phosphorus. The sandy-loam texture of the soil, characterized by good aeration and moisture retention, supports *Actinobacteria* growth and activity^17,19,20^.

### Isolation of *Actinobacteria*

In the current study, *Actinobacteria* were isolated using four different culture media (BEN, GLM, GA, and M2). M2 proved to be the most effective medium for isolating *Actinobacteria* compared to Bennett, GLM, and GA (Supplementary Table S2, and Supplementary Fig. S2). This result could be explained by the constituents of the M2 medium, specifically starch and casein^21^. *Actinobacteria* are capable of catabolizing these macromolecules, thus supplying the nutrients necessary for their growth. this medium contains also trace elements required for bacterial growth^21^. which additionally fosters the proliferation of *Actinobacteria***,** facilitating their successful isolation.

### Characteristics of the SCJ isolate in cultural, micro-morphological, biochemical and physiological terms

The results of the cultural and phenotypic characteristics of the SCJ *Actinobacteria* isolate are shown in Supplementary Table S3, and Supplementary Fig. S3. Based on the specific culture media used, the colonies of the SCJ isolate exhibit circular, punctiform, or star-like forms. Within the ISP media variants, the aerial mycelium of the SCJ isolate assumes a powdery white appearance on all ISP media, while the mycelium on the substrate displays diverse hues such as yellowish, brown, and violet. After growth of the *Actinobacteria* isolate for ten days on ISP1 medium, the blade was observed under a light microscope in the fresh state (magnification =  × 400), and after Gram staining (magnification =  × 1000), and the results are shown in the Supplementary Fig. S3D–F. Under light microscopy, it was discerned that this particular isolate corresponds to a Gram-positive filamentous bacterium with a shape similar to that of *Actinobacteria*. In addition, this isolate consists of a dense, thick mycelial substrate. It is also composed of branched, unfragmented Gram-positive filaments (Supplementary Fig. S3). In terms of hyphal morphology, the SCJ isolate exhibits hyphae that disperse freely. Notably, SCJ isolate produced a violet-brown melanoid pigment on ISP2, ISP4, ISP5 and GYEA media (Supplementary Fig. S3C). The SCJ isolate grows in a pH range from 4.63 to 10.03, with an optimal growth at pH 8.28, and is unable to grow at pH below 4.63 (Table 1). The *Actinobacteria* SCJ isolate exhibits tolerance to NaCl concentrations ranging from 1 to 5%, yet its growth is inhibited beyond 7%. Results from the carbon source test (Table 1) indicate that the *Actinobacteria* isolate is capable of assimilating the various carbohydrate compounds as carbon source, except for ribose. According to Shirling et al.^22^, the identification of *Actinobacteria* is mainly based on morphological features, which are considered stable characters. On the other hand, Williams et al.^23^, demonstrated that certain genera of *Actinobacteria* such as *Streptomyces*, *Streptoverticillium*, *Micromonospora*, *Microbispora*, can be identified from other genera (*Nocardia*, *Actinomadura*) through simple microscopic observation. Based on morphological study, the SCJ isolate displays typical *Streptomyces* traits, enabling us to conclude that it belongs to this group. This result aligns with another work realized by Ningthouja et al.^24^. In fact, strains that grow in a wide range of culture media generally display a morphology typical of *Streptomyces*^25^.

**Table 1:** Carbon assimilation, pH, NaCl and temperature tolerance of SCJ isolate.

Test	Result
Carbon assimilation	
Ribose	−
Melezitose	+
Mannitol	3 +
Trehalose	3 +
Cellobiose	3 +
Sucrose	+
Raffinose	+
Xylose	+
Melibiose	3 +
Mannose	3 +
Fructose	+
Galactose	+
Maltose	3 +
Glucose	3 +
pH tolerance	
4.63	−
5.33	2 +
6.41	2 +
7.31	2 +
8.28	3 +
9.27	2 +
10.03	2 +
NaCl tolerance	
1%	3 +
2%	3 +
3%	3 +
4%	3 +
5%	2 +
7%	−
10%	−
Temperature	
Growth on 4 °C	−
28 °C	3 +
37 °C	+
46 °C	−

( −) no growth; ( +) low growth; (2 +) intermediate growth; (3 +) good growth.

The SCJ strain produced a violet-brown pigment on ISP2, ISP4, ISP5, and GYEA media. These pigments result from the conversion of tyrosine into DOPA-melanin, which imparts the color^26^. The synthesis of these melanoid pigments constitutes a significant trait for the categorization of *Streptomyces*^22^. Some *Actinobacteria* can produce dark-brown substances in culture media, commonly known as melanoid pigments. Vinarov et al.^26^ considered melanoid pigment compounds to be irregular, dark-brown polymers formed by many microorganisms via fermentative oxidation^27^. Moreover, melanoid pigments possess a broad spectrum of biological activities, including radioprotective, antioxidant, antimicrobial, and anti-tumoral functions^28^.

The SCJ strain grows in a pH range from 5.33 to 10.03, with optimal growth at pH 8.28, and is unable to grow at pH below 5.33. Consequently, our observations indicate a preference for neutral to slightly alkaline pH conditions for this strain's growth. Most *Actinobacteria* species are mesophilic, with optimal growth temperatures between 25 and 30 °C^29^. The optimum growth recorded for this strain is between 28 and 37 °C, with no growth at 4 °C or 46 °C. These results agree with those reported by Singh et al.^30^.

The SCJ strain tolerates NaCl concentrations of 1 to 5%, but no growth above 7%. Tian et al.^31^ concluded that saline or hypersaline environments require special attention, as they could offer new avenues for the discovery of new natural and industrially useful molecules. Other studies have demonstrated that *Actinobacteria* have developed adaptations to survive in saline environments^32^, but may be sensitive to osmotic shocks^33^. It is, therefore, important to note that the effect of salinity on the growth of *Actinobacteria* could vary according to the specific species and environmental conditions. The carbon source test showed that the SCJ strain is able to assimilate and use all carbon substrates except ribose. Carbon assimilation by *Actinobacteria* strains is a major step in identification. Pandey et al.^34^ have shown that certain carbon sources are necessary for optimal antibiotic production.

### Molecular identification and phylogenetic approach of the isolate SCJ

**Figure 1:**
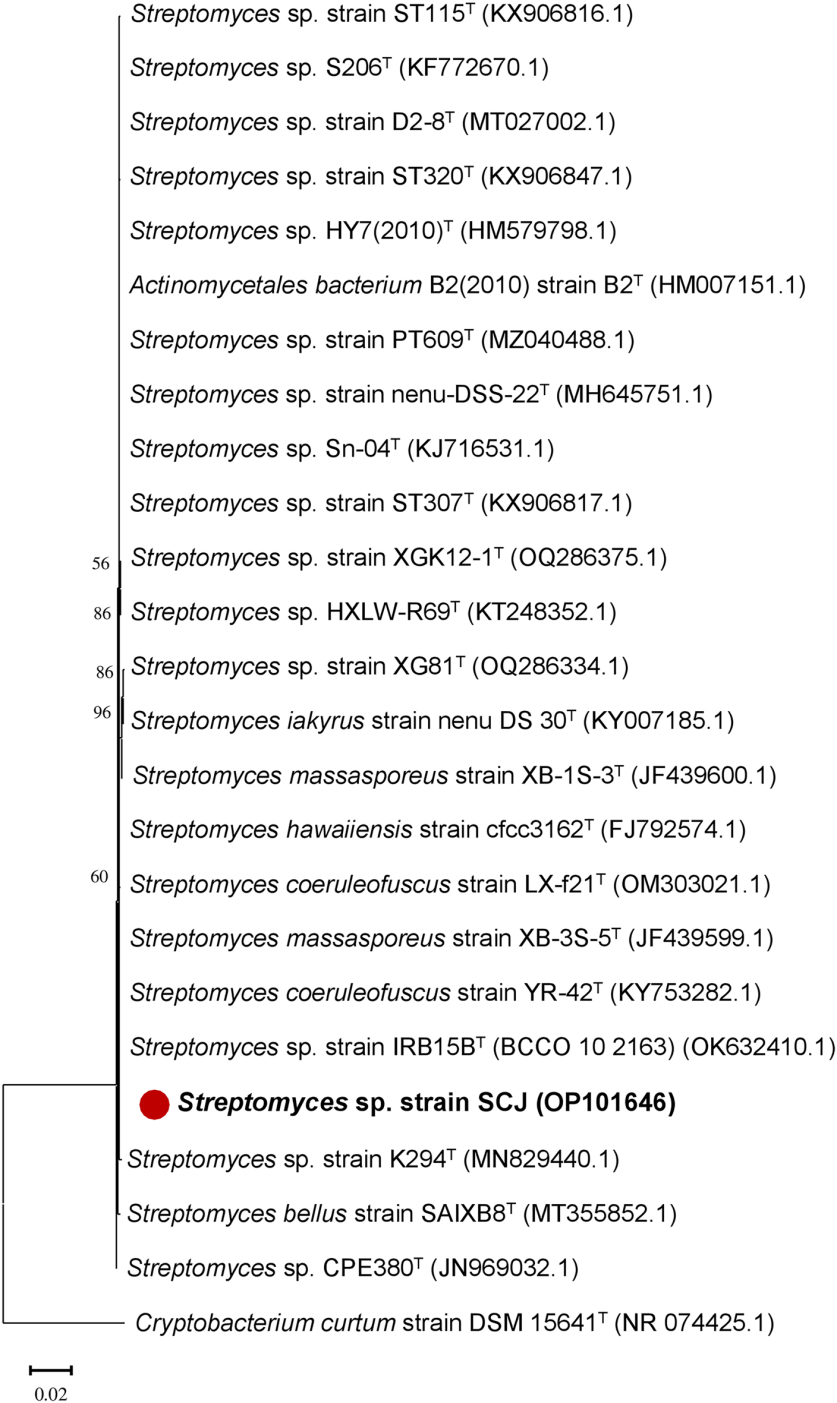
Phylogenetic tree (16S rRNA gene) showing the evolutionary relationship between *Streptomyces* sp. SCJ and its closest known taxa using MEGA X. The bar (0.02) represents the number of substitutions per nucleotide position (1% divergence between sequences). The tree was generated using 1000 bootstraps and GenBank accession numbers are enclosed in parentheses. *Cryptobacterium curtum* was the outgroup in the analysis. *T* Type strain.

The *Actinobacteria* isolate underwent 16S RNA gene extraction and sequencing for phylogenetic approach. DNA was quantified using a NanoDrop spectrophotometer, and electrophoresis confirmed successful amplification (Supplementary Fig. S4). The sequence of the SCJ isolate has been deposited in the GenBank of the National Center for Biotechnology Information (NCBI) https://www.ncbi.nlm.nih.gov/nucleotide/) under the following accession number: OP101646 (Supplementary Table S4). Comparison of the aligned sequence obtained with those available in the GenBank genomic database was used to determine the taxonomic status of this *Actinobacteria* isolate. The results indicate that this isolate belongs to the genus *Streptomyces* (100%). The SCJ isolate showed a very high percentage of similarity at species level (> 99%). Specifically, this isolate showed 99.78% similarity to *Streptomyces coeruleofuscus* YR-42 (KY753282.1), as demonstrated in Supplementary Table S4, and Fig. 1. Nevertheless, despite an exhaustive examination of morphological, micro-morphological, physiological, and biochemical attributes, a definitive classification of the SCJ isolate within specific genera and its resemblance to established species necessitates phylogenetic investigations based on 16S rRNA gene sequencing. The results of molecular identification, based on 16S ribosomal RNA homology, are in agreement with previous studies^35,36^. These findings suggest that the *Streptomyces* genus could be the dominant one in soils compared to other *Actinobacteria* genera. In the study conducted by Kitouni et al.^35^, it was noted that 93% of the active *Actinobacteria* strains were attributed to the *Streptomyces* genus. This suggests that *Streptomyces* is a prominent constituent of the *Actinobacteria* population in the studied environments and highlights its prevalence in soils.

### Primary screening of the SCJ isolate according to culture medium

**Figure 2:**
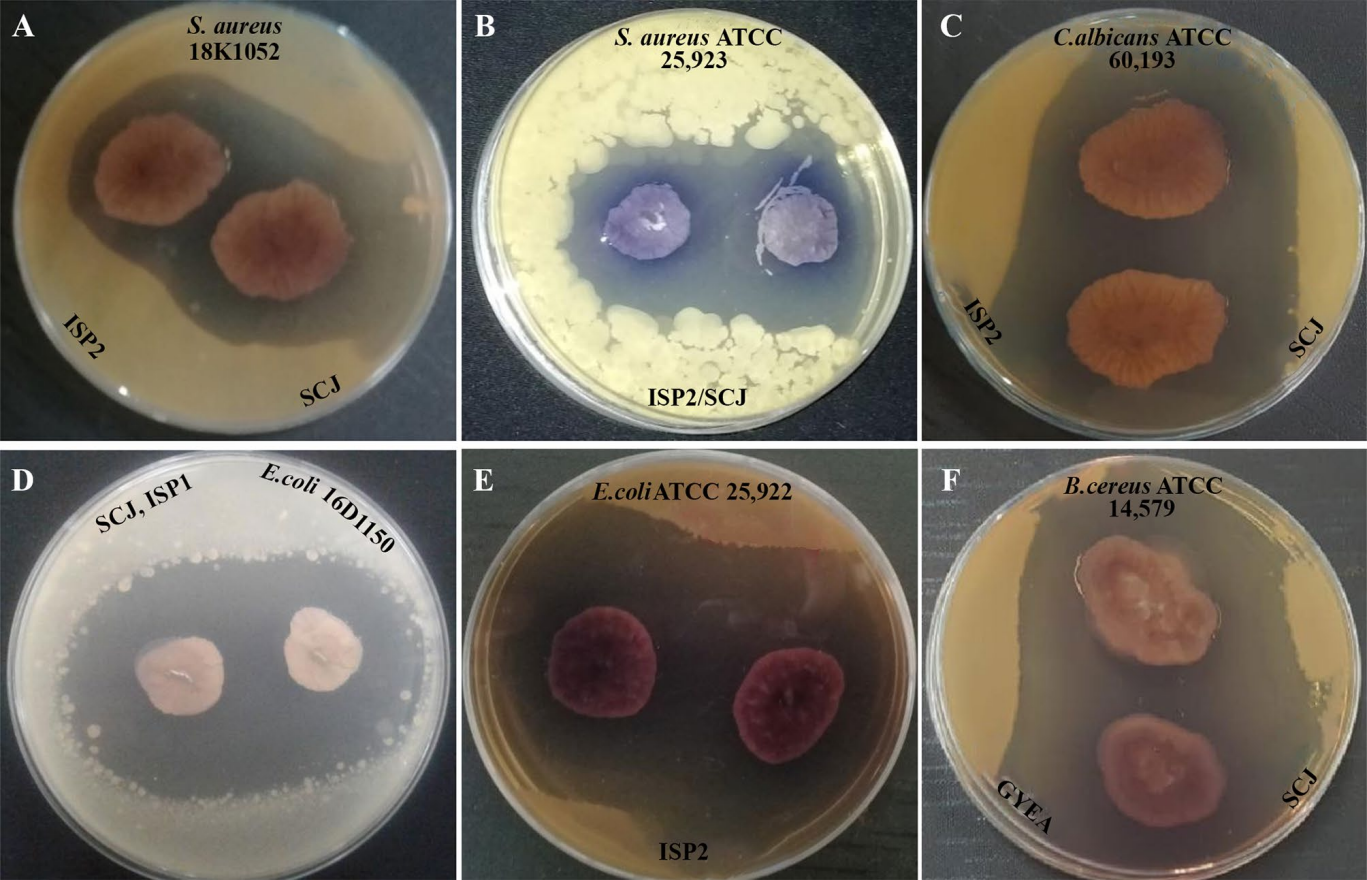
Antimicrobial activity using double-layer method against: (**A**) 18K1052 (clinical *Staphylococcus aureus)*, (**B**) *Staphylococcus aureus* ATCC 25,923, (**C**) *Candida albicans* ATCC 60,193, (**D**) 16D1150 (clinical *Escherichia coli*), (**E**) *Escherichia coli* ATCC 25,922, (**F**) *Bacillus cereus* ATCC 14,579.

Colonies displaying the macroscopic and microscopic morphological characteristics of *Actinobacteria* were chosen for assessing their antimicrobial activities against 14 target microorganisms using the double-layer method (Table 2, Fig. 2). To evaluate the impact of culture medium on secondary metabolite production by *Actinobacteria* strains, we selected four widely recognized media (ISP1, ISP2, Bennett, and GYEA). These media were chosen based on their established efficacy for antimicrobial activity production^37,38^. The inclusion of these diverse media in our study aimed to investigate how variations in culture conditions, influenced by distinct media formulations, affect the biosynthesis of secondary metabolites. The results obtained are presented in Table 2. The highest level of antimicrobial activity was observed on ISP2, followed by GYEA, ISP1, and finally Bennett. Indeed, statistical analysis of variance for the results (implemented as an ordinary one-way ANOVA with Tukey's multiple comparisons test) revealed significant disparities among these media for the SCJ isolate. The SCJ isolate exhibited moderate to robust antimicrobial activity against all the five fungi isolates tested (*Candida albicans* ATTC 60,193, *Fusarium* sp. MN944571, *Fusarium* sp. MN944573, *Fusarium* sp. MN944574, and *Fusarium* sp. MN944577). In addition to its antifungal effects, the SCJ isolate displayed potent antagonistic properties against the majority of both Gram-positive bacterial strains (*Bacillus cereus* ATCC 14,579, *Staphylococcus aureus* ATCC 25,923, *Staphylococcus aureus* CECT 976, clinical *Enterococcus* 18k1386, *Staphylococcus aureus* 18k1052, and clinical *Staphylococcus epidermidis* 16C1181), and Gram-negative bacteria (*Escherichia coli* ATCC 25,922, *Pseudomonas aeruginosa* ATCC 27,853, clinical *Enterobacter* 18K794, clinical *Proteus mirabilis*, clinical *Proteus vulgaris* 16C1737, clinical *Neisseria gonorrhoeae* 16C1170, clinical *Escherichia coli* 16D1150). The antimicrobial activity of the *Streptomyces sp*. strain was remarkably high on the tested agar media (Bennett, ISP1, ISP2, and GYEA), with the highest production observed on ISP2, followed by GYEA, ISP1, and Bennett. Moreover, considering the large zones of inhibition observed, ISP2 was selected as the optimal medium for secondary metabolite production. This choice is further supported by similar findings reported in other studies^39,40^.

**Table 2:** Primary screening (antimicrobial activity of SCJ isolate) by the double-layer method on four culture media (Bennett, ISP1, ISP2 and GYEA).

Test strains	SCJ isolate			
	Bennett^c^	ISP1^c^	ISP2_2_^a^	GYEA^b^
*Escherichia coli* ATCC 25,922	−	38 ± 2.16	56.33 ± 4.50	−
*Staphylococcus aureus* CECT 976	25.5 ± 4.5	31 ± 1	31 ± 0.82	30.33 ± 2.87
*Staphylococcus aureus* ATCC 25,923	−	31.67 ± 3.40	37.33 ± 1.89	51.5 ± 0.5
*Bacillus cereus* ATCC 14,579	−	31 ± 2.16	57.68 ± 4.11	51.67 ± 3.86
*Pseudomonas aeruginosa* ATCC 27,853	−	−	34.67 ± 4.11	30.67 ± 1.70
Clinical* Enterococcus*	28.33 ± 1.70	−	−	30.33 ± 3.09
Clinical* Enterobacter*	−	27.5 ± 3.5	45 ± 2.5	38.67 ± 2.87
Clinical *Staphylococcus aureus*	25.5 ± 4.5	31 ± 1	31 ± 0.82	30.33 ± 2.87
Clinical *Proteus mirabilis*	32 ± 2.16	−	32 ± 1.63	−
Clinical *Proteus vulgaris*	−	−	41 ± 0.82	−
Clinical *Neisseria gonorrhea*	34.67 ± 2.49	29 ± 5.10	49.67 ± 4.50	40.33 ± 1.25
Clinical *Escherichia coli*	−	38 ± 2.16	56.33 ± 4.49	−
Clinical* Staphylococcus epidemidis*	17.5 ± 0.71	9.5 ± 0.71	19 ± 1.41	29 ± 1.41
*Candida albicans* ATTC 60,193	−	−	57 ± 2.16	−
*Fusarium* sp. MN944571	−	−	26.5 ± 0.71	−
*Fusarium* sp. MN944573	−	−	24.5 ± 0.71	−
*Fusarium* sp. MN944574	−	−	31 ± 1.41	−
*Fusarium* sp. MN944577	−	−	41 ± 1.41	−

(-) no inhibition zones.

a, b, c, and d indicate significant differences between media for each test strain (p < 0.0001).

The results highlight the significant influence of culture medium composition on the production of secondary metabolites by the *Streptomyces* sp. strain. Specifically, the antimicrobial activity was markedly enhanced when using in the ISP2 medium (which is rich in malt extract, suggesting that the components in malt extract played a pivotal role in augmenting this antimicrobial activity). It is also possible that the high glucose content of the Bennett media (10 g/L compared to 4 g/L in ISP2) played a detrimental role. In this context, previous studies have demonstrated that high concentrations of glucose exert a catabolic repressive effect on antibiotic production, but not on macrolides^39,41^. The ISP1 medium, lacking glucose entirely, appears to be a poor production medium, as indicated by its low antimicrobial activities. This could be attributed to the absence of glucose, which serves as a rapidly assimilable carbon source for most antibiotic-producing microorganisms. Indeed, the synthesis of secondary metabolites by *Actinobacteria* heavily relies on the formulation of the culture medium, with a specific emphasis on the carbon and nitrogen sources^30^.

### Production kinetics of secondary metabolites of the isolate SCJ

**Figure 3:**
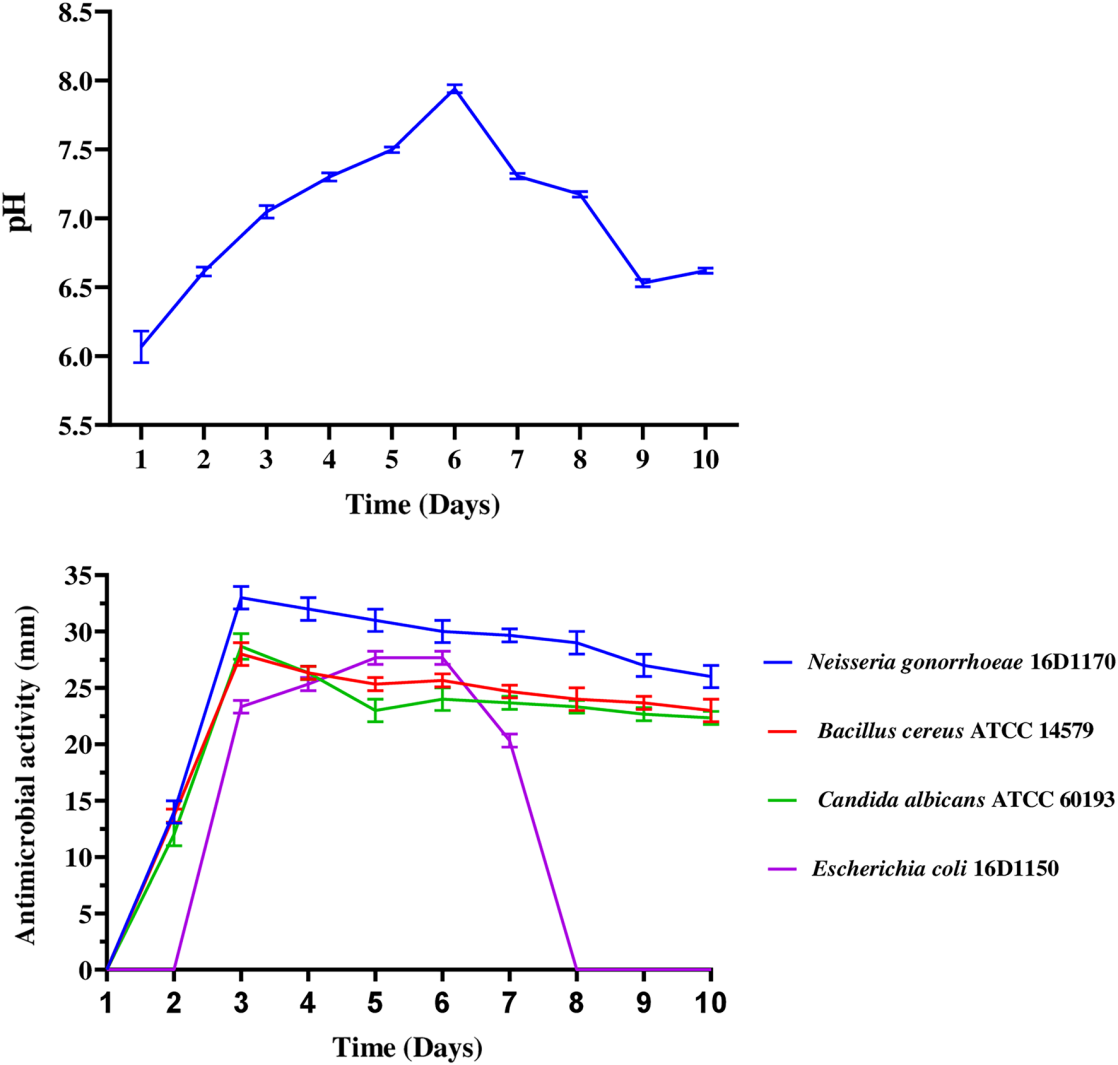
Kinetics of pH evolution, and antimicrobial activity in ISP2 medium, against *Neisseria gonorrhoeae* 16D1170, *Bacillus cereus* ATCC 14,579, *Candida albicans* ATCC 60,193 and *Escherichia coli* 16D1150. Antimicrobial activity was measured using the well technique. The experiments were performed in triplicate.

The aim of assessing the kinetics of secondary metabolite production in the SCJ strain was to identify the day when the concentration of metabolites reaches its maximum. This ensures that the extraction process is aligned with the growth phase associated with maximum productivity. The outcomes of the secondary metabolite production kinetics, and pH fluctuations, carried out in liquid ISP2 medium, are shown in Fig. 3, and Supplementary Fig. S5. Throughout the incubation period, pH kinetics exhibited slight variation, ranging between 5.67 and 8.01. This pH fluctuation can be attributed to the degradation of organic nitrogen sources, such as amino acids in yeast extracts and peptones in malt extracts, which undergo deamination, releasing ammonium^42^. This latter accumulates in the medium over time, leading to increased alkalinity of the medium during the incubation period^42^.

The Streptomyces sp. strain SCJ reaches its highest production of secondary metabolites on days 5 and 6 post-incubation, beginning during the stationary phase. The kinetics of secondary metabolite production reveal multiple peaks, indicating that this strain produces a wide range of substances. These substances might either be derivatives of one another or belong to distinct groups with potentially different activity spectra^43^.

In *Streptomyces* species, the initiation of antibiotic production typically aligns with the start of the stationary phase in liquid cultures^44^. When subjected to stress, such as nutrient depletion, a portion of the mycelium undergoes autolysis through programmed cell death, releasing nutrients that facilitate the formation of aerial hyphae and spores. The initiation of sporulation is linked to the production of secondary metabolites^45^. This production can commence either during the exponential^40^ or the decline phase^46^.

### Secondary screening of the SCJ isolate according to the organic solvent

**Figure 4:**
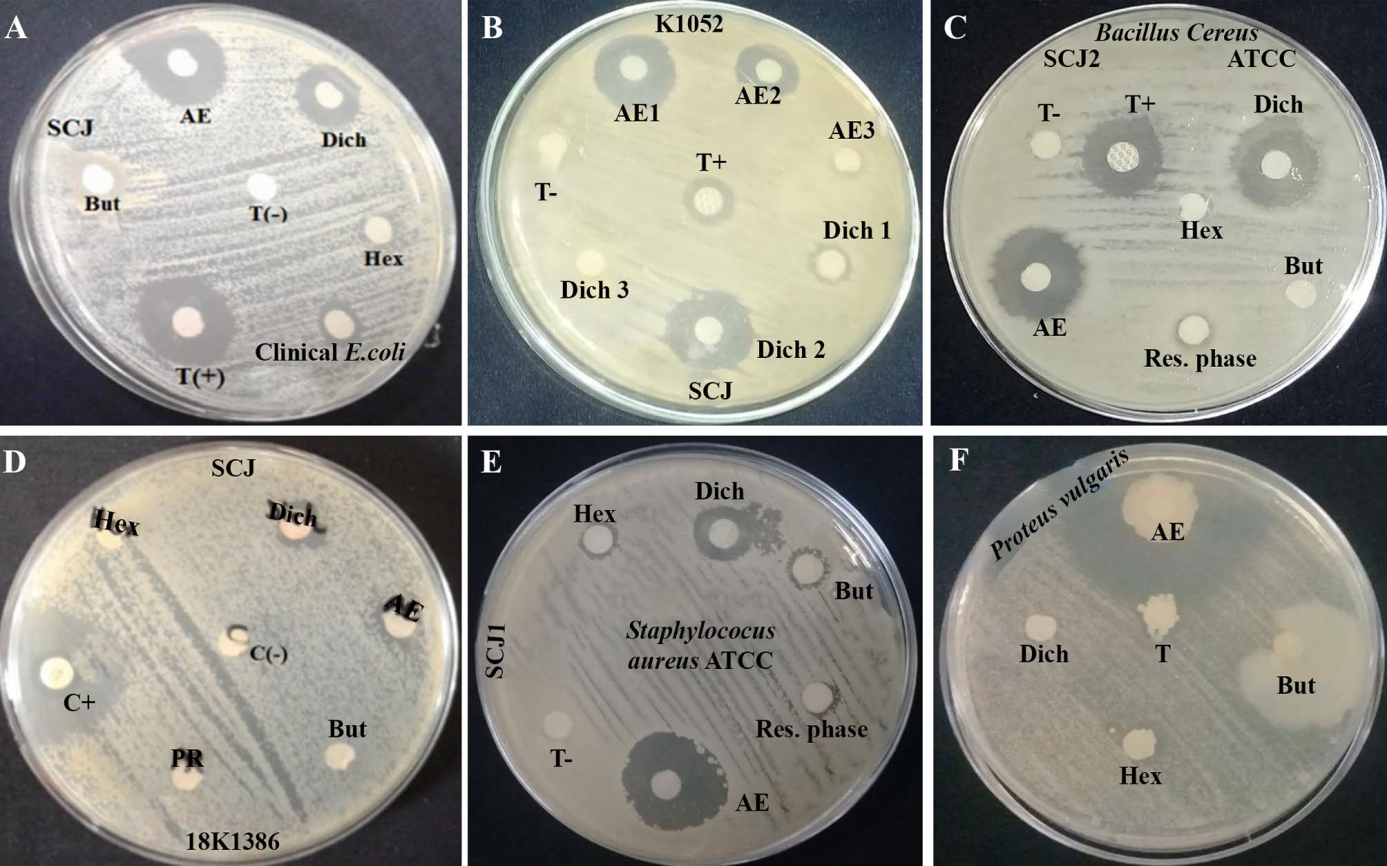
Antimicrobial activity using disk diffusion method against (**A**) clinical *Escherichia coli*, (**B**) clinical *Staphylococcus aureus*, (**C**) *Bacillus cereus* ATCC 14,579, (**D**) clinical *Enterococcus*, (**E**) *Staphylococcus aureus* ATCC 25,923, (**F**) clinical *Proteus vulgaris*. T( +): Positive control (Streptomycin), T( −): Negative control, *Hex* Hexane, *Dich* Dichloromethane, *AE* Ethyl acetate, *But* Butanol, *PR* Residual phase.

The extraction of secondary metabolites from the *Actinobacteria* SCJ isolate was conducted based on the findings derived from growth kinetics and the results obtained during the primary screening (choice of medium). The production kinetics were conducted in the liquid ISP2 medium, which was chosen as the optimal medium for secondary metabolite production during the primary screening. The culture was terminated after five days of agitation-based incubation, a duration determined to correspond to the optimal day for secondary metabolite production as established through the kinetics study. The SCJ isolate was subjected to secondary screening against certain multi-resistant pathogenic bacteria (MDR), as well as some phytopathogenic fungi. Table 3 displays the antimicrobial activities extracted using n-hexane, dichloromethane, ethyl acetate, and n-butanol. Our aim was to capture a diverse spectrum of secondary metabolites from *Actinobacteria* strains. Employing solvents with varying polarities allowed us to explore the polarity-dependent distribution of secondary metabolites within the extracts. Through optimization, we determined that ethyl acetate and dichloromethane were the most effective solvents for extracting the main bioactive metabolites. Precisely, the antimicrobial activities of the SCJ isolate were highest in the ethyl acetate and dichloromethane extracts (Table 3, Fig. 4, and Supplementary Fig. S6). The results of this secondary screening revealed that the SCJ isolate was more active against Gram-positive bacteria than Gram-negative bacteria. The greatest zones of inhibition were witnessed within the organic phase, indicating that the active secondary metabolites produced by this isolate were hydrophobic and more easily extractable by polar (ethyl acetate) and medium-polar (dichloromethane) phases. No antimicrobial activity was observed in the aqueous phase, further supporting the notion that the secondary metabolites secreted by this isolate are hydrophobic and better extracted with organic solvents. Within the ethyl acetate extract, a majority of the tested microorganisms displayed susceptibility to the predicted compounds generated by the SCJ isolate. Regarding anti-candidosis and antibacterial activity, the ethyl acetate extract of the SCJ isolate exhibited notably larger zones of inhibition in comparison to both the positive control and the other extracts. This could be attributed to the higher solubility of secondary metabolites in the ethyl acetate solvent^47^.

**Table 3:** Secondary screening (antibacterial activity of SCJ isolate) using the disc diffusion method according to extraction solvents.

Test strains	SCJ isolate				
	PC^a^	Hex^c^	Dich^c^	EA^b^	But^c^
*Staphylococcus aureus* ATCC 25,923	27.5 ± 2.12	8.5 ± 0.71	12 ± 0	26.5 ± 0.71	8.5 ± 0.71
*Bacillus cereus* ATCC 14,579	28 ± 4.24	9.5 ± 0.71	18 ± 2.83	23 ± 2.83	–
*Pseudomonas* ATCC 27,853	28.5 ± 3.54	–	–	–	–
*Escherichia coli* ATCC 25,922	28 ± 1.41	–	11.5 ± 2.12	10 ± 1.41	–
Clinical *Enterococcus*	11 ± 1.41	–	13 ± 1.41	16 ± 1.41	–
Clinical* Staphylococcus aureus*	24 ± 1.41	–	20.5 ± 2.12	22 ± 2.83	–
Clinical* Proetus vulgaris*	35.5 ± 0.71	–	19 ± 1.41	14.5 ± 0.71	–
Clinical* Neisseria gonorrhea*	25.5 ± 2.12	–	–	9.5 ± 0.71	–
Clinical* Escherichia coli*	27 ± 1.41	–	18 ± 2.83	19.5 ± 2.12	–
Clinical* Klebsiella pneumoniae*	26 ± 1.41	–	–	15 ± 1.41	5.5 ± 2.12
*Candida albicans* ATTC 60,193	27 ± 2.83	–	15.5 ± 3.54	13 ± 1.41	–
*Fusarium* sp. MN944577	31.5 ± 071	–	–	33 ± 2.83	–
*Fusarium* sp. MN944567	28.5 ± 2.12	–	–	9.5 ± 0.71	–
*Fusarium* sp. MN944568	27 ± 1.41	–	–	18.5 ± 2.12	–
*Fusarium* sp. MN944569	27.5 ± 0.71	–	–	9.5 ± 0.71	–
*Fusarium* sp. MN944570	26.5 ± 0.71	–	–	13 ± 0	–

Values expressed are means ± SD.

*PC* Positive control (Streptomycin), *Hex* Hexane, *Dich* Dichloromethane, *EA* Ethyl acetate, *But* Butanol, " − " no zone of inhibition.

a, b, and c indicate significant differences between the different extract for each test strain (p < 0.05).

### UV–visible spectrum analyses of the SCJ isolate crude organic extract

The absorption spectrum of the ethyl acetate extract derived from the SCJ strain obtained does not show absorption peaks characteristic of polyene molecules, which are characterized by three characteristic absorption maxima in the UV–visible between 291 and 405 nm^46^. Only two absorption peaks were observed, occurring at wavelengths of 300 and 301 nm (Supplementary Fig. S7). Because polyene molecules are known to be toxic, the statement highlights an interesting discovery. While polyene antifungal molecules demonstrate efficacy against fungi, they tend to engage with cholesterol due to its structural resemblance to ergosterol, the primary sterol present in fungal cells; this interaction with cholesterol potentially elucidates the toxicity associated with polyene antifungal molecules^48^. According to Yilma et al.^48^, the toxicity of polyene antifungals has been studied and established in previous research. Understanding this mechanism is critical for drug development and choosing safe and effective antifungal compounds for medical applications.

### Identification of bioactive compounds present in the crude ethyl acetate organic extract of the SCJ isolate using GC–MS

**Figure 5:**
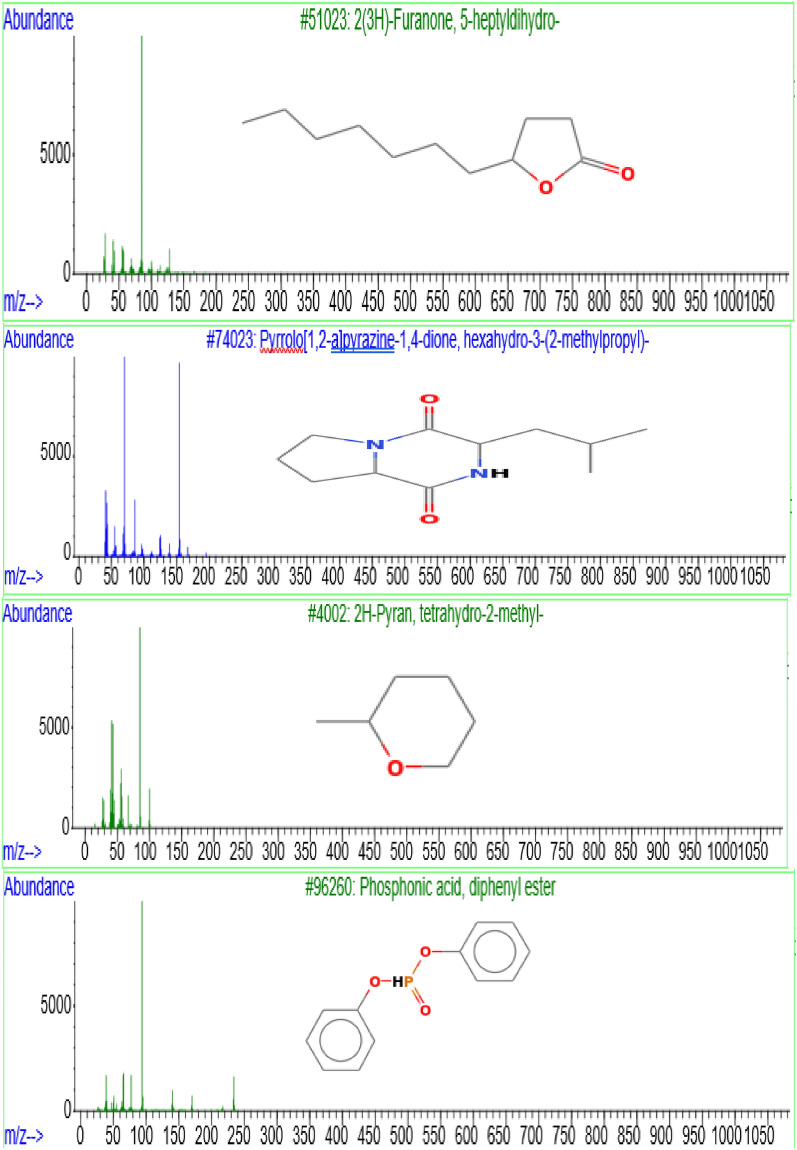
GC–MS spectra of 4 important secondary metabolites present in ethyl acetate extract of SCJ strain.

The GC–MS analysis of the ethyl acetate extract derived from *Streptomyces* sp. SCJ strain revealed the presence of nine volatile predicted compounds eluting between 13.130 and 27.238 min. Of these predicted compounds, three including 3,5-Dimethylpyrazol; pyrrolizidine derivatives (Pyrrolo[1,2-a]pyrazine 1,4-dione, hexahydro-3-(2-methylpropyl)-); and Phosphonic acid, diphenyl ester (Table 4, Supplementary Fig. S8, and Fig. 5), each possessing a spectrum of diverse biological activities ranging from insecticidal and antitumor effects to analgesic, antimicrobial, and antioxidant properties^49,50^. It is possible that all the compounds recorded in the ethyl acetate extract derived from the *Streptomyces* sp. SCJ strain tested are responsible for all the biological activities exhibited by this extract. In addition, all the biological activities shown by *Streptomyces* sp. SCJ may have been caused by the synergistic effect of secondary metabolites present in the ethyl acetate extract of this strain.

**Table 4:** Volatile compounds identified by GC–MS from ethyl acetate crude extract of *Streptomyces* sp*.* SCJ strain.

RT (time)	Area (%)	M.W (g/mol)	Molecular formula	Compound name	Reported bioactivity
13.130	1.71	94.20	C_2_H_6_S_2_	Disulfide, dimethyl	Antioxidant, Antifungal, Analgesic effect^51^
13.242	0.51	249.18	C_12_H_10_O_4_P^-^	Phosphonic acid, diphenyl ester	Anticancer drugs or pesticides, antibacterial agents, neuroactive compounds, whose possible applications range from medicine and agriculture^50^
13.491	5.97	96.13	C_5_H_8_N_2_	3,5-Dimethylpyrazole	Insecticidal and antitumor activities, analgesic, anti-inflammatory, antimicrobial, anticonvulsant,antidepressant, antimycobacterial, antioxidant, antiviral^52^
14.313	0.67	254.35	C_12_H_18_N_2_O_2_S	Piperazine, 1-methyl-4-[2-(p-tolylsulfonyl) ethyl]-	Anthelminthic drugs^53^
15.992	0.57	282.4	C_17_H_30_O_3_	Tetrahydropyran Z-10-dodecenoate	Antibacterial against several micro-organisms, including Gram negative and Gram-positive bacteria^10^
16.127	2.87	100.16	C_6_H_12_O	2H-Pyran, tetrahydro-2-methyl-	Not yet reported
16.995	0.22	250.26	C_12_H_17_F_3_O_2_	Trifluoroacetyl-lavandulol	Antibacterial activity^54^
17.423	3.43	184.27	C_11_H_20_O_2_	2(3H)-Furanone, 5-heptyldihydro-	Antifungal Activity^55^
27.238	1.35	210.27	C_11_H_18_N_2_O_2_	Pyrrolo[1,2-a] pyrazine 1,4-dione, hexahydro-3-(2-methylpropyl)-	Insecticidal^56^ and Fungicidal activity^57^. Antifungal, Antioxidant, and Antibacterial^58^

*RT* retention time, *M.W* molecular weight.

### Evaluation of the toxicity of SCJ ethyl acetate extract by a hemolysis test on normal human cells (red blood cells)

**Figure 6:**
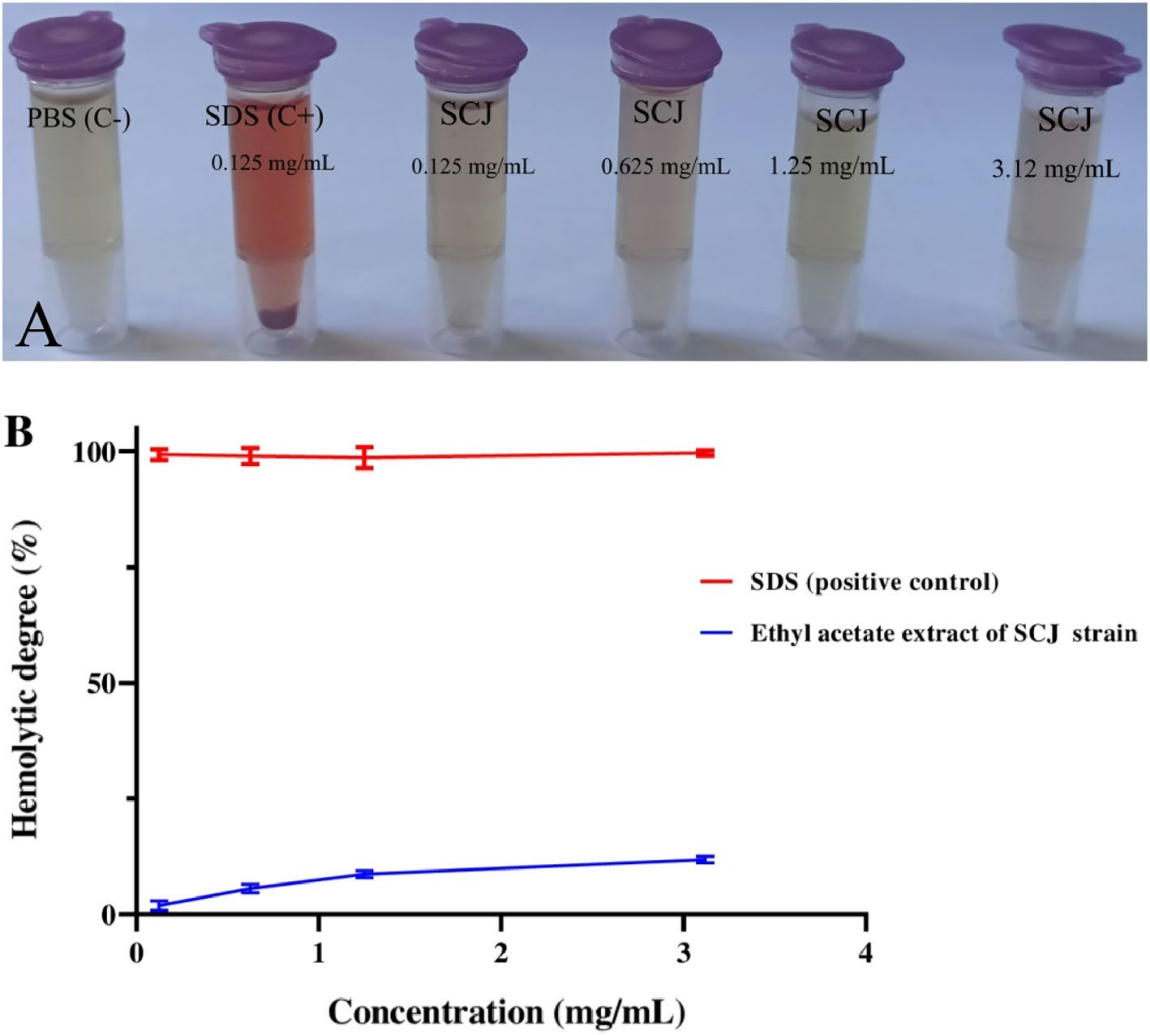
Evaluation of hemolytic activity of SCJ ethyl acetate extract on red blood cells. (**A**) hemolysis test, (**B**) UV–vis spectroscopy. The experiment was performed in triplicate.

The ethyl acetate extract derived from *Streptomyces* sp. strain SCJ has demonstrated a notable absence of erythrocyte hemolysis at the tested concentrations (Fig. 6A,B). Quantitative analysis through UV–vis spectroscopy further confirmed the minimal hemolytic activity of this extract **(**Fig. 6B**)**. These findings from our study suggest that the ethyl acetate extract obtained from *Streptomyces* sp. strain SCJ can be deemed safe for human use, as it exhibited no adverse effects on human erythrocytes. According to Saurav & Kannabiran^59^, it is crucial to assess membrane stability when introducing new drugs, and the use of red blood cells as a model for such stability assessments proves highly valuable.

## Conclusion

This work highlights the potential of *Streptomyces* as a valuable source of natural bioactive compounds. The isolated *Streptomyces* strain from an unexplored terrestrial habitat in north-eastern Morocco produced powerful secondary metabolites, particularly with antibacterial and antifungal properties. The ethyl acetate extract displayed significant antimicrobial activity against drug-resistant bacteria and phytopathogenic fungi. The absence of polyene molecules and the hemolytic activity against erythrocytes in the extract are promising. Our GC–MS analysis identified nine bioactive predicted compounds with anticarcinogenic, antioxidant, and antimicrobial properties. Further researches are required to understand the intracellular mechanisms of these compounds, along with their identification and confirmation using advanced techniques such as fourier transform infrared spectroscopy (FTIR), high-performance liquid chromatography (HPLC), high-resolution mass spectrometry (HRMS) and reconfirming structure using nuclear magnetic resonance (NMR).

## Materials and methods

### Collection of soil samples and isolation of *Actinobacteria*

The SCJ strain was isolated from a soil sample collected from the Northwestern Morocco site located in Titt Mellil garden belonging to the Casablanca-Settat region of Morocco (33°33′14.1"N, 7°29′05.4"W), during February and early March 2019. Briefly, 10 g of the soil sample was crushed, sieved and mixed with 90 mL of sterile distilled water. The resulting suspension was vigorously vortexed for 30 min and supernatant was decanted. Afterward, the supernatant was serially diluted to 10^–4^, and 100 µL of each dilution was spread on four *Actinobacteria* isolation media; (1) M2^60^, (2) GA^60^, (3) GLM^25,61^ and (4) Bennett^62^. After sterilization and cooling, each medium was supplemented with 50 mg/L of actidione (Cycloheximide) to inhibit fungal growth. Subsequently, Petri dishes were incubated at 28 °C and observed daily for a period ranging from one to three weeks. Colonies of *Actinobacteria*, distinguishable by their macroscopic and microscopic characteristics, were subcultured onto ISP2 medium using the streak method to obtain pure cultures. Ultimately, the resulting pure colonies were preserved at 4 °C on ISP2 agar for short-term storage and at − 20 °C in an ISP2 suspension containing 20% glycerol for long-term storage^63^.

### Physico-chemical analyses of the soil sample

The pH of the soil sample was determined using a calibrated pH meter (OCRISON, micro-pH 2000). The electrode was immersed in a suspension with a with a soil/distilled water ratio of 2/5 (g/mL). Electrical conductivity (EC) was measured using conductivity meter (sevenGoTM). Soil texture was assessed through sedimentation technique. Total nitrogen content was quantified using the Dumas method^64^. Organic matter (OM) was analyzed using UV–visible spectrophotometer technique^65^. Chemical elements including C, O, Mg, Si, Fe, K and Ca were analyzed by scanning electron microscope (SEM) (JEOL: JSM-IT500HR)^66^. Additionally, elements such as Zn, Mn, Cl, Al, P, Cu and S were analyzed by the energy dispersive X-ray fluorescence method (Epsilon 3XLE from PANalytical, France).

### Soil sample pretreatment

To enhance the abundance of *Actinobacteria*, the soil sample underwent pretreatment via two approaches: firstly, by air-drying at room temperature for a duration of 7 days^67^; secondly through an enrichment method which consists of taking 10 g of the previously dried and sieved soil sample, mixing it with 1 g of calcium carbonate (CaCO_3_), and subsequently incubating it at room temperature (25 °C) for 7 to 9 days^35^.

### Cultural, micro‑morphological, biochemical and physiological characteristics of the SCJ isolate

In accordance with a previous study by Shirling et al.^22^, cultural traits such as growth extent, medium pigmentation, colony appearance, and the occurrence of diffusible pigments within the agar were noted on ISP (International *Streptomyces* Project) media (ISP1, ISP2, ISP4, ISP5, and GYEA). All these media were seeded by the streak technique, followed by incubation in Petri dishes at 28 °C. Daily checks were conducted over a period of 10 days.

The micro-morphological characters of the pure SCJ isolate were determined using a light microscope equipped with a digital camera (Olympus CX43RF) in the fresh state and after Gram staining by the coverslip culture technique^68^. Briefly, a sterile lamella was gently introduced into the solid ISP1 medium at a 45° angle. A drop of bacterial suspension was then placed against the lamella in contact with the surface of the medium. After 10 days of incubation at 28 °C, the slide was carefully removed from the agar and placed on a blade and observed under a light microscope at different magnifications^68^.

Physiological and biochemical characters were assessed based on previous studies^69,70^. These studies encompass melanoid pigment production, tolerance to varying NaCl concentrations (1%, 2%, 3%, 4%, 5%, 7%, and 10%), pH tolerance (4.63, 5.33, 6.41, 7.31, 8.28, 9.27, and 10.03), temperature-dependent growth (4 °C, 28 °C, 37 °C, and 46 °C), and the utilization of carbohydrates and their derivatives as the exclusive carbon source.

### Genotypic identification

For genotypic identification, 16S rRNA sequencing was employed to confirm the genus classification of the SCJ isolate. Bacterial DNA was extracted using the Mag Purix Bacterial DNA Extraction Kit following the manufacturer's protocol (MagPurix, Manufacturer). DNA concentration and purity were assessed using a NanoDrop 8000 spectrophotometer by calculating the 260/280 and 260/230 ratios (Thermo Scientific, Manufacturer). Extracted DNA was subsequently stored at − 20 °C until further use. Amplification of the gene encoding 16S rRNA from *Actinobacteria* strains was performed in an ABI "Verity" thermal cycler using the universal bacterial primers Fd1 (5'-AGAGAGTTTGATCATGGCTCAG-3') and rP2 (5'-ACGGTTACCTTGTTACGACTT-3') described by Weisburg et al.^71^ to obtain an amplicon size of 1500 bp under the following reaction conditions: Initial denaturation at 95 °C for 2 min followed by 35 cycles at 95 °C for 30 s, 52 °C for 30 s and 72 °C for 30 s and a final extension at 72 °C for 3 min. The amplified products were analyzed by electrophoresis. Specifically, 8 μL of PCR products were plated on an agarose gel (1%) in the presence of the 1-kb molecular weight marker (thermo scientific). The image was visualized by the G-Box photo Gel-Documentation system.

All amplified products obtained were sequenced to validate their identities. Sequencing reactions were performed using a Big Dyeterminator version 3.1 cycle sequencing kit (Applied Biosystems) using the same primers as those used for PCR amplification. The nucleotide sequence was analyzed using software available on the Internet at the National Center for Biotechnology Information (http://www.ncbi.nlm.nih.gov). The works of extraction, amplification and sequencing were carried out at the National Center for Scientific and Technological Research (CNRST), Morocco. Nucleotide sequences were aligned with MEGA X software^15^ using MUSCLE alignment. The phylogenetic tree was constructed with the same alignment software using the neighbor-joining tree method^15^.

### Primary screening of the SCJ isolate of *Actinobacteria*

To determine the optimal medium for antibiotic production, the antimicrobial potency of the SCJ isolate was assessed using the double-layer method^38^. This was conducted on four solid media (ISP1, ISP2, GYEA, and Bennett) against various microorganisms, including *Escherichia coli* ATCC 25,922, *Staphylococcus aureus* ATCC 25,923, *Bacillus cereus* ATCC 14,579, *Candida albicans* ATCC 60,193 (these microoganisms were collected from the Institut Pasteur Casablanca Morocco), as well as 12 phytopathogenic fungi identified as *Fusarium* species with the following accession numbers: MN944566, MN944567, and so on, up to MN944577 (these phytopathogenic fungus were obtained from Laboratory of Agro-Alimentary and Health, Faculty of Sciences and Techniques, Hassan First University of Settat Moroco). In the double-layer approach, the *Actinobacteria* isolate was applied at the center of each medium (approximately 15–20 mm in diameter) and incubated at 28 °C for 10 days. Overlying the cultures, a 5 mL layer of weakly agarized Muller Hinton (MH) medium (8 g agar/L) was introduced, previously inoculated with the target microorganism. Following solidification of the second layer, the Petri dishes were incubated again at 37 °C for 24 h for bacteria and 48 h for fungi. Antimicrobial activity was evaluated by measuring the zones of inhibition in millimeters (mm). The phytopathogenic fungi (*Fusarium* sp.) were isolated from the mango tree *Mangifera indica* L. (infected at leaf level) native to the Indo-Burma region (senegal), is one of the oldest cultivated fruit trees in the world^72^.

## Secondary metabolite production kinetics

To pinpoint the optimal duration for secondary metabolite production, a 10-day kinetic study was conducted using ISP2 broth medium, which had been selected during the primary screening phase. Initially, a loopful of mature *Actinobacteria* culture from the SCJ isolate (grown for 10 days) was introduced into a 250 mL Erlenmeyer flask containing 50 mL of ISP2 broth. After two days of continuous agitation (28 °C, 150 rpm), 5 mL of each pre-culture was extracted to inoculate separate 250 mL Erlenmeyer flasks, each containing 50 mL of the medium. These flasks were then subjected to continuous agitation for 10 days. The daily evolution of antimicrobial activity over this period was assessed using the Muller Hinton agar (MHA) diffusion method (well method), wherein 50 μL of culture supernatant was added into wells of 6 mm diameter^40,46^. This was performed against *Bacillus cereus* ATCC 14,579 and *Candida albicans* 60,193. Additionally, changes in pH were monitored throughout this period following the approach outlined by Pfefferle et al.^73^.

## Fermentation and extraction of secondary metabolites

The *Actinobacteria* SCJ isolate was cultivated in liquid medium (ISP2). After fermentation, liquid–liquid extraction using increasingly polar organic solvents (hexane, dichloromethane, ethyl acetate, and n-butanol) was performed. In 500 mL Erlenmeyer flasks with 100 mL of ISP2 broth, the isolate was cultured at 28 °C with continuous shaking at 150 rpm. The agitation duration was optimized for maximal metabolite production, determined from production kinetics. After centrifugation at 10,000 g for 20 min, supernatant was mixed with each solvent sequentially. Organic extracts were vacuum-evaporated in a rotary evaporator (Buchi B-491 heating bath) (below 45 °C) to preserve metabolites. Residues were dissolved in dimethylsulfoxide (DMSO) to calculate their initial concentrations^70,74^.

### Evaluation of antimicrobial activity

The SCJ organic extract's antimicrobial activity was assessed using the disc diffusion method as reported by Badji et al.^38^. Initial testing involved five plant pathogenic fungi (*Fusarium* sp. MN944568, *Fusarium* sp. MN944569, *Fusarium* sp. MN944570, *Fusarium* sp. MN944571, and *Fusarium* sp. MN9445677). Subsequently, the extract's efficacy was tested against five multi-resistant clinical strains (*Escherichia coli* 16D1150, *Enterococcus faecalis* 18K1386, *Staphylococcus aureus* 18K1052, *Proteus vulgaris* 16C1737, and *Neisseria gonorrhoeae* 16D1170), obtained from the Pasteur Settat medical analysis laboratory. For antifungal evaluation, fungi were subcultured on potato extract agar (PDA) medium and incubated at 28 °C for 10 days. Inoculum optical densities were standardized between 0.18 and 0.20 using a spectrophotometer (Selectra VR2000, Barcelona, Spain) at 623 nm, corresponding to approximately 10^6^ spores/mL^75^. Sterile 6-mm-diameter disks were loaded with 20 μL of extract, and allowed to dry for approximately 15 min using a Bunsen burner, and then placed on the surface of MHA medium that had been previously inoculated with microorganisms using the swabbing technique. Fungal strains were grown on potato extract agar (PDA). Negative controls featured DMSO-soaked disks of equal volume, while positive controls involved the antibiotics Streptomycin and Cycloheximide for antibacterial and antifungal assessments, respectively. Following 2 h at 4 °C for diffusion, inhibition zone diameters were measured in mm after 24 h of bacterial incubation at 37 °C and 48 h of fungal incubation at 28 °C.

### UV–visible analysis of SCJ extract

Absorption spectra of the DMSO-solubilized SCJ extract were recorded between 190 and 850 nm using a scanning UV–vis spectrophotometer (HACH lange DR6000)^76^.

### GC–MS analysis of organic extracts

The volatile compound profile of The SCJ extract was characterized using gas chromatography (GC) (Agilent 7890A Series) combined with mass spectrometry (MS) equipped with a multimode injector and HP-5MS column with a dimension of 30 m × 0.250 mm × 0.25 μm. This analysis was conducted at the Moroccan Foundation for Advanced Science, Innovation and Research (MAScIR) Institute. A volume of 4 µL of the extract, solubilized in an adequate volume of DMSO, was injected into the column using a 1:4 split mode with helium serving as the carrier gas at a flow rate of 1.7 mL/min. The ion source and quadrupoles were maintained at temperatures of 230 °C and 150 °C, respectively. The oven temperature program initiated at 60 °C and concluded at 360 °C. Compound identification was performed by comparing the obtained mass spectra with data available in the NIST MS 2017 library^76,77^.

### Evaluation of hemolytic activity by hemolysis assay

The hemolytic activity of the SCJ organic extract was assessed using a hemolysis assay employing human red blood cells (RBCs), following the methods reported by Rajendran et al.^78^ and Zhu et al.^79^,with some modifications. A volume of 2 mL whole blood was combined with 4 mL of phosphate-buffered saline (PBS) and subjected to centrifugation at 9000 g for 5 min at 24 °C. The resulting supernatant was discarded, and the pellet was washed twice with 10 mL of PBS, then ultimately diluted to 20 mL using PBS. Afterward, 400 µL of the diluted RBC suspension was mixed with 1.6 mL of different concentrations of the SCJ organic extract, ranging from 0.125 to 3.12 mg/mL, while PBS served as the negative control and Sodium Dodecyl Sulfate (SDS) as the positive control. The experimental setup was duplicated. Tubes were incubated at 37 °C for 1 h and subsequently centrifuged for 5 min at 5000 g, with optical density (OD) measurements taken at 540 nm. The extent of hemolysis was represented as the hemolysis rate, computed using the subsequent formula:

$$Hemolysis \;rate= \left[\frac{\left(OD \left(Test\right)- OD \left(Negative \;control\right)\right)}{OD \left(Positive \;control\right)- OD \left(Negative \;control\right)}\right]x \;100 \%$$

### Statistical analysis

Data from all experiments were repeated at least twice and results were expressed as Mean ± standard deviation. An ordinary one-way ANOVA followed by Tukey's multiple comparisons test was performed using GraphPad Prism 8.4.3 software (GraphPad sotftware Inc., San Diego, CA, USA) to test for significant differences between groups in the antimicrobial activity test. The results were considered statistically significant when p ≤ 0.05.

### Supplementary Information

Supplementary Information.

## Data Availability

The datasets generated during and/or analysed during the current study are available from the corresponding author on reasonable request. Genomic sequence of *Streptomyces* sp. SCJ has been deposited at the National Centre for Biotechnology Information (NCBI) GenBank (https://www.ncbi.nlm.nih.gov/nucleotide/) under the following accession number OP101646.
