# Supplementary material for: Antimicrobial potential of Streptomyces coeruleofuscus SCJ isolated from microbiologically unexplored garden soil in Northwest Morocco
Source: Sci Rep. 2024 Feb 9;14:3359. doi: 10.1038/s41598-024-53801-x (PMC10858231; doi:10.1038/s41598-024-53801-x)
Supplement: Supplementary file 1 — Supplementary Information. [file 41598_2024_53801_MOESM1_ESM.docx]

**Antimicrobial potential of *Streptomyces coeruleofuscus* SCJ isolated from microbiologically unexplored garden soil in Northwest Morocco**

**Said Rammali^a,^*,** Abdellatif Rahim^b^, Mohamed El Aalaoui^c^, Khadija Dari^a^, Bouchaib Bencharki^a^, Aicha Habach^d^ , Lamiri Abdeslam^e^, Abdelkrim khattabi^a^

^a^Laboratory of Agro-Alimentary and Health, Faculty of Sciences and Techniques, Hassan First University of Settat, B.P. 539, Settat 26000, Morocco

^b^Laboratory of Biochemistry, Neurosciences, Natural Ressources and Environment, Faculty of Sciences and Techniques, Hassan First University of Settat, B.P. 539, Settat 26000, Morocco

^c^Regional Center of Agronomic Research of Settat, Tertiary Road 1406, At 5 Km from Settat,

Settat 26400, Morocco

^d^Biotechnology Unit, National Institute of Agronomic Research of Rabat, Rabat, Morocco, Av. Annasr, Rabat 10000

^e^Applied Chemistry & Environment Laboratory, Faculty of Sciences and Techniques, Hassan First University of Settat, B.P. 539, Settat 26000, Morocco

* Corresponding authors:

[rammali_fst@hotmail.fr](mailto:rammali_fst@hotmail.fr) / [s.rammali@uhp.ac.ma](mailto:s.rammali@uhp.ac.ma) (Said RAMMALI)

**SUMMARY**

1. General methods

[1.1 Recommended media for *Actinobacteria* isolation](#_Toc107464915) 1

1.2 Recommended media for testing antimicrobial activity............................................................1

1.3 Recommended medium for testing NaCl tolerance...................................................................1

1.4 Recommended medium for the production of melanin pigment.................................................1

1.5 Other media used in this study....................................................................................................2

**2. Supplementary figures**

Figure S1. Scanning electron microscope (SEM) analysis of minerals...........................................3

Figure S2. *Actinobacteria* isolated on M_2_ medium……………………….......................................4

Figure S3. Microscopic and morphological characterization of the SCJ isolate following incubation on various media, including melanoid pigment production on GYEA(C) medium......5

Figure S4. Agarose gel electrophoresis for amplification of genes coding for 16S rRNA of *Streptomyces* sp***.*** SCJ strain …*…………………………………………………………………………….*6

Figure S5. Kinetics of secondary metabolite production by the SCJ strain against *Bacillus* *cereus* ATCC 14579………………………………………………………………………………………7

Figure S6. Antifungal activity of SCJ isolate using disk diffusion method against A: *Fusarium* sp. MN944568, B: *Fusarium* sp. MN944570, C: *Fusarium* sp. MN944577, D: *Candida* *albicans* ATTC 60193……...………………………………………..……...................................................8

Figure S7. UV-visible spectra of the crude ethyl acetate extract of *Streptomyces* sp. SCJ…………9

Figure S8. GC-MS of Ethyl acetate crude extract of *Streptomyces* sp. strain SCJ with peaks indicating the presence of bioactive compounds (according to the NIST database) ……………10

**3. Supplementary tables**

Supplementary Table 1. Physico-chemical analysis of soil samples...............................................11

Supplementary Table 2. Total number of *Actinobacteria* isolates recovered from site A according to the culture media used................................................................................................................12

Supplementary Table 3. Morphological and cultural characteristics of a SCJ *Actinobacteria* isolate using solid ISP media.....................................................................................................................13

Supplementary Table 4. Molecular identification of the selected SCJ *Actinobacteria* isolate based on 16S rRNA gene sequencing…………………………………………………….......................14

References.…………………………………………………….....................................................15

**1. General methods**

**1.1 Recommended media for *Actinobacteria* isolation**

M2: 10 g of starch, 0.3 g of casein, 2 g of KNO_3,_2 g of NaCl, 0.05 g of MgSO_4_,7H_2_O, 2 g of K_2_HPO_4,_0.02 g of CaCO_3_, 0.01 g of FeSO_4_, 7H_2_O, 1 g of glucose, 15 g of agar, 1L distilled water, and pH adjusted at 7. 2^1^.

GA: 10 g of Glucose, 0.5 g of asparagine, 0.5 g of K_2_HPO_4_, 15 g of agar, 1L distilled water, and pH adjusted at 6^1^.

GLM: 10 g of glucose, 3 g of yeast extract, 3 g of malate extract, 5 g of tryptone, 15 g of agar, 1L distilled water, and pH adjusted at 7.2^2,3^.

Bennett : 10 g of glucose, 2 g of yeast extract, 1 g of meat extract, 2 g of tryptone, 15 g of agar, 1L distilled water, and pH adjusted at 7.2^4^.

**1.2 Recommended media for testing antimicrobial activity**^5^

ISP1: 3 g of yeast extract, 5 g of tryptone, 16 g of agar, 1L of distilled water, and pH adjusted at 7.15.

ISP2: 4 g of yeast extract, 4 g of glucose, 10 g of malate extract, 16 g of agar, 1L of distilled water, and pH adjusted at 6.51.

GYEA: 10 g of yeast extract, 10 g of glucose, 16 g of agar, 1L of distilled water, and pH adjusted at 6.96.

Bennett: 10 g of glucose, 2 g of yeast extract, 1 g of meat extract, 2 g of tryptone, 15 g of agar, 1L distilled water, and pH adjusted at 7.2.

**1.3 Recommended medium for testing NaCl tolerance**

YEA medium: 3 g of yeast extract, 5 g of peptone, 15 g of agar, 1L of distilled water, and pH adjusted at 7.2.

**1.4 Recommended medium for the production of melanoid pigment**

ISP9: 2.64 g of (NH_2_) SO_4_, 2.38 g of KH_2_PO_4_, 5.65 g of K_2_HPO_4_, 1 g of MgSO_4_, 7H_2_O, 20 g of agar, and 1L of distilled water.

100 mL salt solution: 4 g of CuSO_4_, 5H_2_O, 1.1 g of FeSO_4_, 7H_2_O, 7.9 g of MnCl_2_, 4H_2_O, 1.5 g of ZnSO_4_, 7H2O.

GYEA: 10 g of yeast extract, 10 g of glucose, 16 g of agar, 1L of distilled water, and pH adjusted at 6.96.

**1.5 Other media used in this study**

PDA medium: 4 g of potato extract 20 g of glucose, 15 g of agar, 1L distilled water, and pH adjusted at 7.5.

Muller Hinton Agar medium: Ready to use

CLED medium: Ready to use.

**2. Supplementary figures**


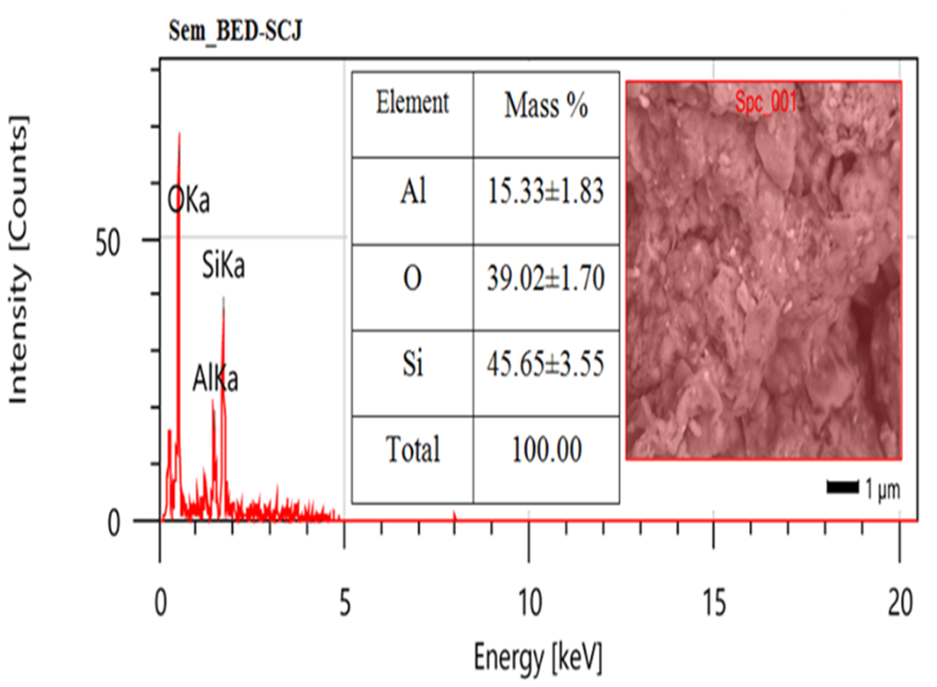


**Figure S1.** Scanning electron microscope (SEM) analysis of minerals


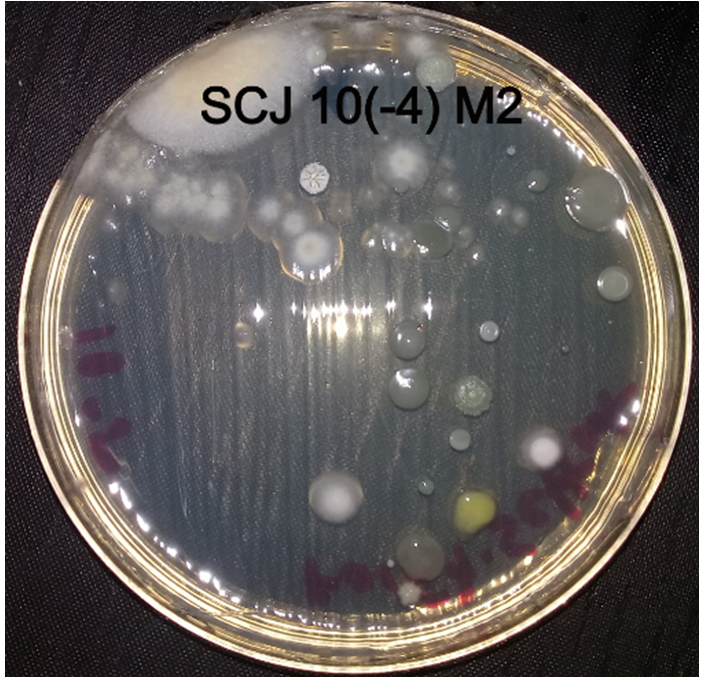


**Figure S2. *Actinobacteria*** isolated on M_2_ medium


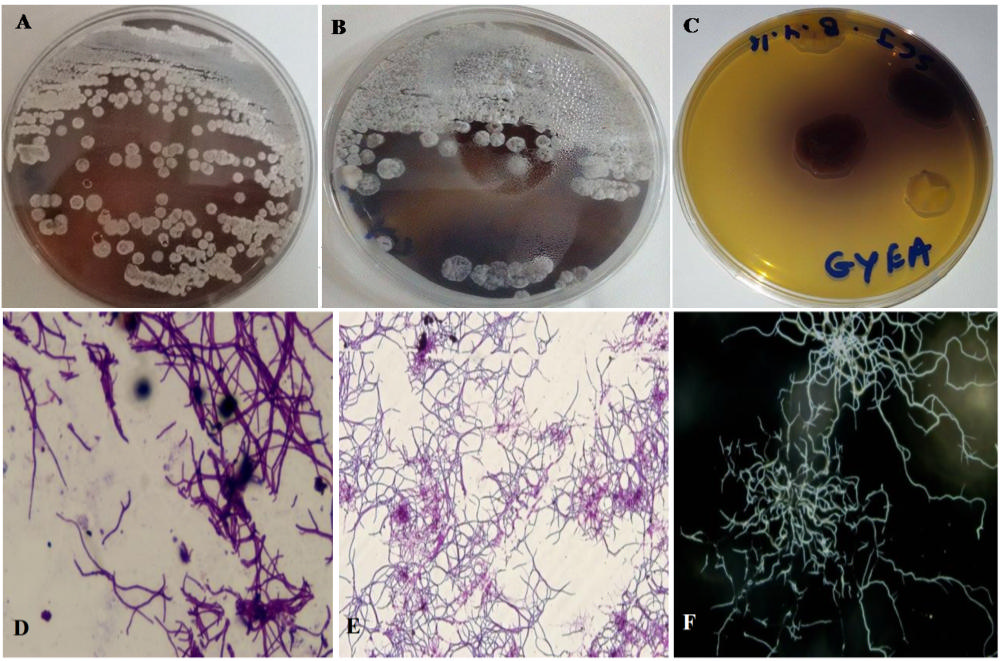


**Figure S3.** Microscopic and morphological characterization of the SCJ isolate following incubation on various media, including melanoid pigment production on GYEA(C) medium





Figure S4. Agarose gel electrophoresis for amplification of genes coding for 16S rRNA of *Streptomyces* sp*.* SCJ strain

E16: *Streptomyces* sp*.* SCJ strain, E14, E15-E42: other isolates, T-: Negative Control, Bands were visualised by the photo documentation system " G Box ".


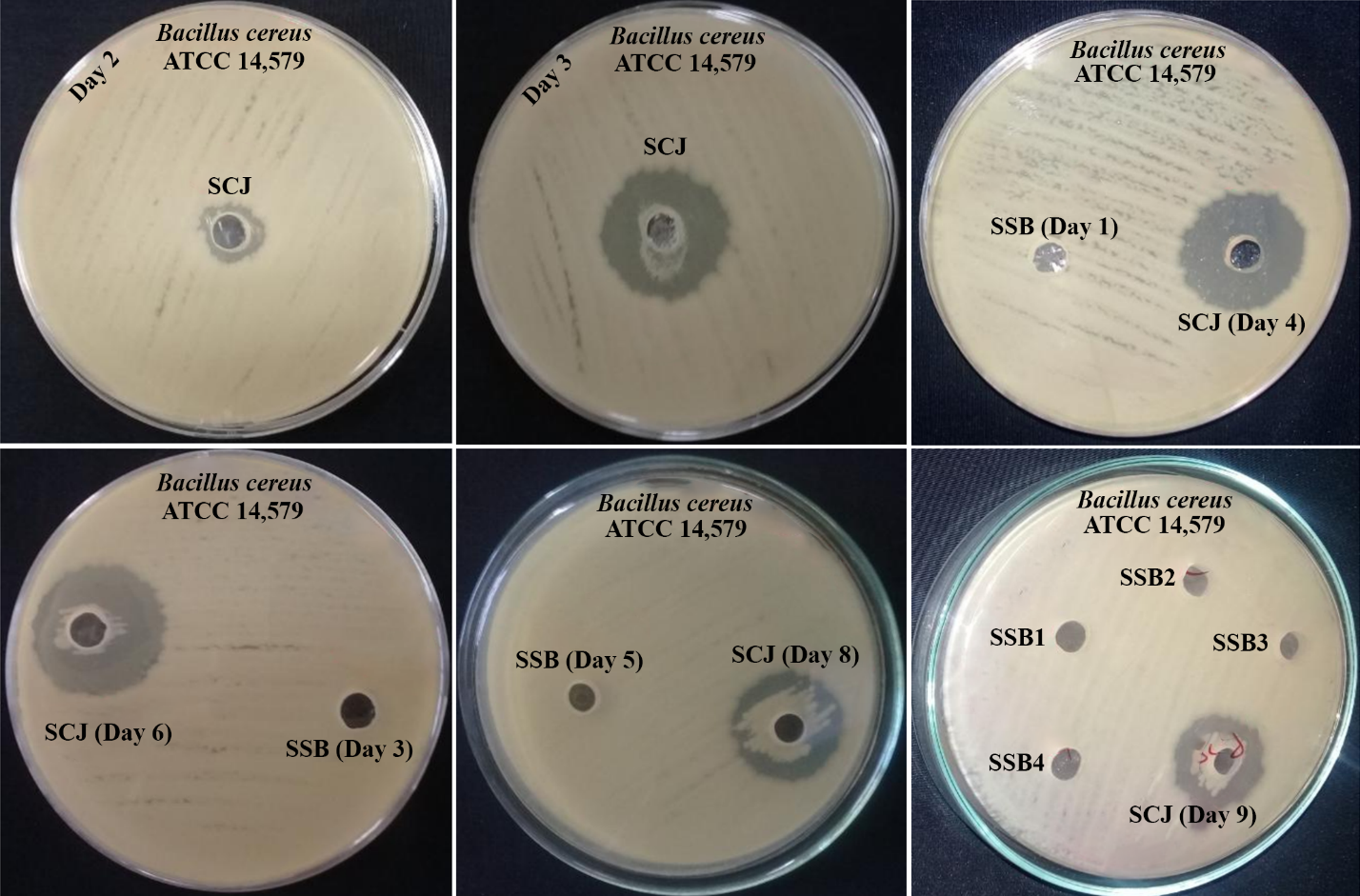


**Figure S5.** Kinetics of secondary metabolite production by the SCJ strain against *Bacillus* *cereus* ATCC 14579


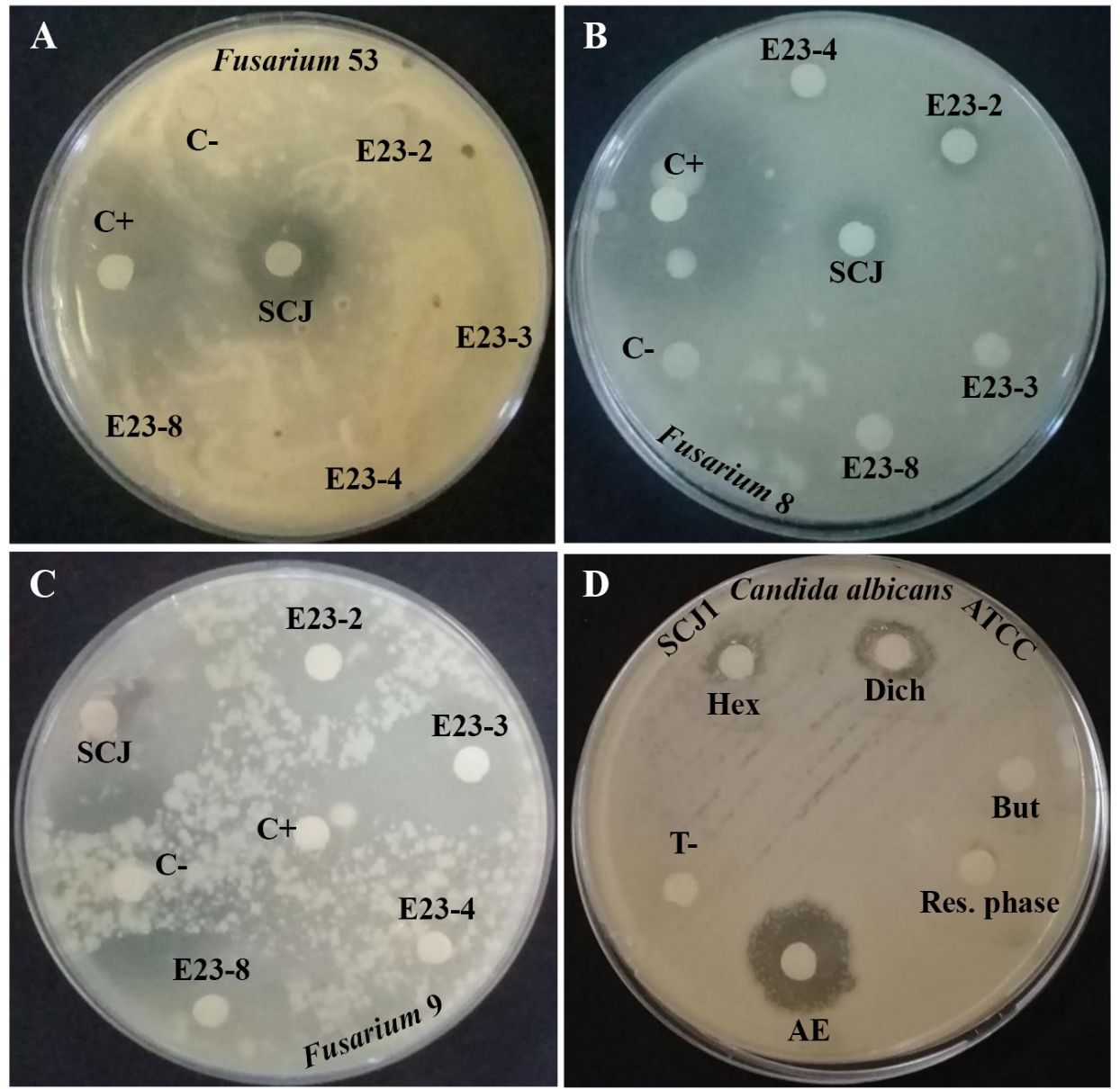


**Figure S6.** Antifungal activity of SCJ isolate using disk diffusion method against **A:** *Fusarium* sp. MN944568, **B:** *Fusarium* sp. MN944570, **C:** *Fusarium* sp. MN944577, **D:** *Candida albicans* ATTC 60193

Figure S7. UV-visible spectra of the crude ethyl acetate extract of *Streptomyces* sp. SCJ


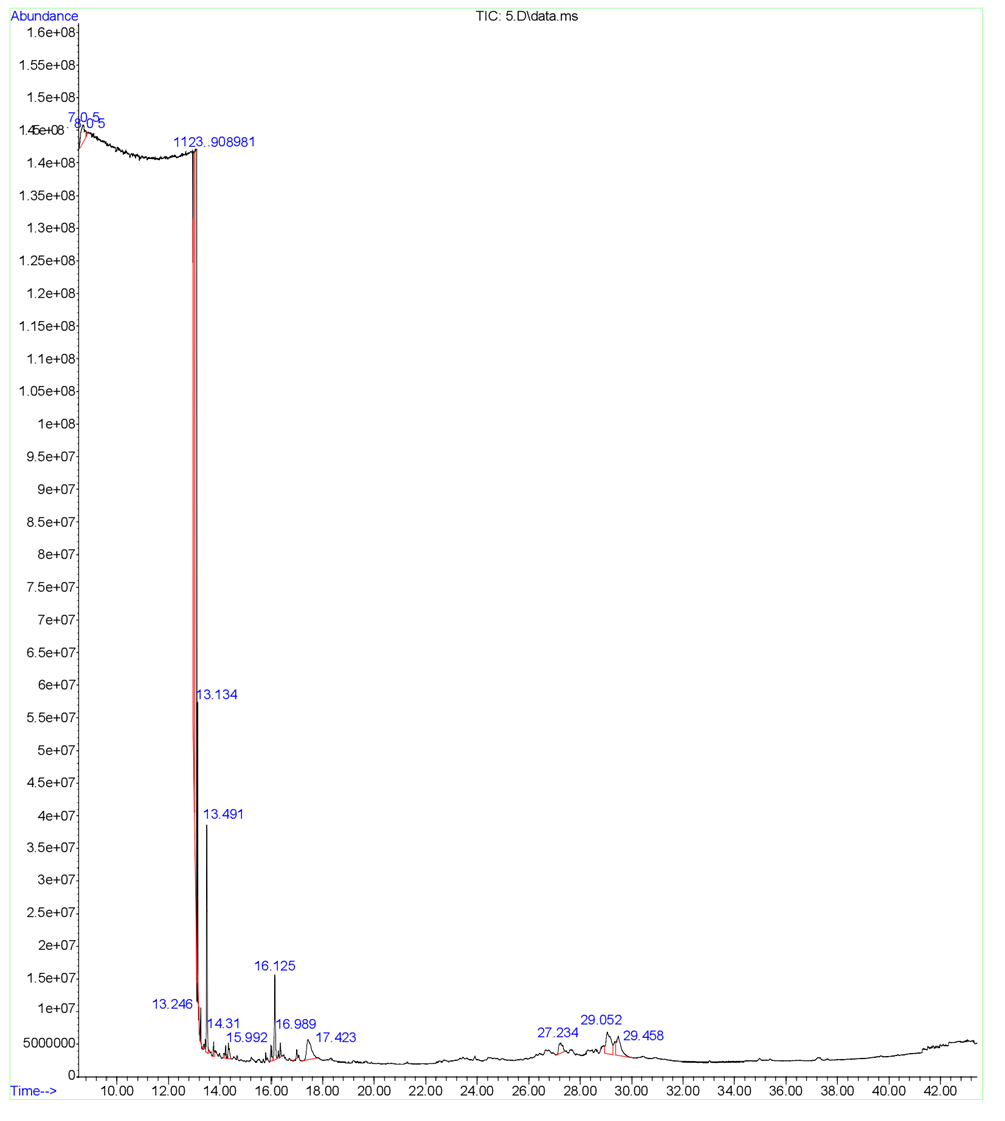


**Figure S8.** GC-MS of Ethyl acetate crude extract of *Streptomyces* sp. strain SCJ with peaks indicating the presence of bioactive compounds (according to the NIST database)

**3. Supplementary tables**

**Supplementary Table S1:** Physico-chemical analysis of soil samples (EC) Electrical

conductivity; (OM) Organic matter; (TN) Total Nitrogen

| Physico-chemical parameters | Site (Tit Mellil Garden Casablanca) |
| --- | --- |
| Textural soil types | Sandy- silty |
| Clay (%) | 5.00 |
| Sand (%) | 75.0 |
| Silt (%) | 19.9 |
| pH | 7.71 |
| EC (ds/m) | 0.32 |
| OM (%) | 2.43 |
| TN (%) | 0.10 |
| Mg (%) | 0.35 |
| K (%) | 1.04 |
| Ca (%) | 14.3 |
| S (%) | 0.87 |
| Cl (%) | 0.51 |
| P (%) | 0.28 |
| Fe (%) | 5.44 |
| Mn (%) | 0.05 |
| Cu (%) | 0.01 |
| Zn (%) | 0.01 |

| Soil collection site | Number of *Actinobacteria* colonies in different isolation media 10^3^ CFU/mL) | | | | Total number of *Actinobacteria* colonies  (10^3^ CFU/mL) | Number of colonies with morphological characteristics in each medium | | | | Number total of isolates |
| --- | --- | --- | --- | --- | --- | --- | --- | --- | --- | --- |
|  | M_2_ | Ben | GLM | GA |  | M_2_ | Ben | GLM | GA |  |
| Site A | 4 | 0 | 0 | 0 | 4 | 1 | 0 | 0 | 0 | 1 |

**Supplementary Table S2:** Total number of *Actinobacteria* isolates recovered from site A according to the culture media used

(Site A) Garden Tit Mellil Casablanca.

**Supplementary Table S3.** Morphological and cultural characteristics of a SCJ *Actinobacteria* isolate using solid ISP media

| *Actinobacteria* isolate | Growing media | Cultural characteristics | | | | |
| --- | --- | --- | --- | --- | --- | --- |
|  |  | Growth | Colony aspect | Aerial mycelium | Substrate mycelium | Diffusible pigment |
| SCJ | ISP_1_ | +++ | Circular | White, powdery, unstarred | Yellowish | Absence |
|  | ISP_2_ | +++ | Circular, irregular | White, powdery | Brown | Brown |
|  | ISP_4_ | +++ | Circular | White, powdery | Violet | Violet |
|  | ISP_5_ | +++ | Punctiform | White, powdery | Pale yellowish | Absence |
|  | GYEA | +++ | Circular | White, starry | Violet-brown | Violet |

(+++) Abondant.

**Supplementary Table S4**. Molecular identification of the selected SCJ *Actinobacteria* isolate based on 16S rRNA gene sequencing

| Isolate Name | Query  Length  (bp) | Nearest known species^a^ | Percent identity  (%) | Identification^b^  (Scientific Name) | NCBI GenBank accession number |
| --- | --- | --- | --- | --- | --- |
| SCJ | 1389 | *Streptomyces coeruleofuscus* YR-42 (T)([KY753282.1](https://www.ncbi.nlm.nih.gov/nucleotide/KY753282.1?report=genbank&log$=nuclalign&blast_rank=1&RID=EJJAYVRM013)) | 99.78 | [*Streptomyces coeruleofuscus*](https://www.ncbi.nlm.nih.gov/Taxonomy/Browser/wwwtax.cgi?id=66879) | OP101646 |

^a^NCBI's nucleotide BLAST program was used to find the closest match against the non-redundant reference RNA sequence database (refseqrna). The strain number, strain type (T), and GenBank accession number are all listed after the species name.

^b^Isolates with a greater percentage of identity than 98.78% and only one closest match for the same identity characteristics are classified up to the species level.

To assess the reliability of a phylogenetic tree, MEGA provides the Bootstrap test. This test uses the bootstrap re-sampling strategy, so you need to enter the number of replicates. For a given data set, applicable tests and the phylogeny inference method are enabled. Neighbor joining has an additional test (Interior Branch), which requires the same input as bootstrap.

**References**

1. Bouaziz, S. *et al.* Antifungal activity of *Streptomyces* sp.14 strain isolated from Ouargla (Southeast of Algeria): identification, production and characterization of the active substance. *Int. J. Biosci.* **9**, 45–56 (2016).

2. Das, R., Romi, W., Das, R., Sharma, H. K. & Thakur, D. Antimicrobial potentiality of *Actinobacteria* isolated from two microbiologically unexplored forest ecosystems of Northeast India. *BMC Microbiol.* **18**, 1–16 (2018).

3. Thakur, D., Yadav, A., Gogoi, B. K. & Bora, T. C. Isolation and screening of *Streptomyces* in soil of protected forest areas from the states of Assam and Tripura, India, for antimicrobial metabolites. *J. Mycol. Med.* **17**, 242–249 (2007).

4. Lee, E. J., Hwang, K. Y., Lee, H. S. & Chung, N. Characterization of a new *Streptomyces* sp. a1022 as a potential biocontrol agent. *J. Appl. Biol. Chem.* **54**, 488–493 (2011).

5. Badji, B., Riba, A., Mathieu, F., Lebrihi, A. & Sabaou, N. Activité antifongique d’une souche d'*Actinomadura* d'origine saharienne sur divers champignons pathogènes et toxinogènes. *J. Mycol. Med.* **15**, 211–219 (2005).

6. Rossi-Tamisier, M., Benamar, S., Raoult, D. & Fournier, P. E. Cautionary tale of using 16s rRNA gene sequence similarity values in identification of human-associated bacterial species. *Int. J. Syst. Evol. Microbiol*. **65**, 1929–1934 (2015).
